# The Role of Genetics and Epigenetic Regulation in the Pathogenesis of Osteoarthritis

**DOI:** 10.3390/ijms241411655

**Published:** 2023-07-19

**Authors:** Kajetan Kiełbowski, Mariola Herian, Estera Bakinowska, Bolesław Banach, Tomasz Sroczyński, Andrzej Pawlik

**Affiliations:** Department of Physiology, Pomeranian Medical University, 70-111 Szczecin, Poland; kajetan.kielbowski@onet.pl (K.K.); mariola.herian@gmail.com (M.H.); esterabakinowska@gmail.com (E.B.); banbol@pum.edu.pl (B.B.); tomasz.sroczynski8@gmail.com (T.S.)

**Keywords:** osteoarthritis, single-nucleotide polymorphism, epigenetic regulation, DNA methylation, histone modification, non-coding RNA

## Abstract

Osteoarthritis (OA) is progressive disease characterised by cartilage degradation, subchondral bone remodelling and inflammation of the synovium. The disease is associated with obesity, mechanical load and age. However, multiple pro-inflammatory immune mediators regulate the expression of metalloproteinases, which take part in cartilage degradation. Furthermore, genetic factors also contribute to OA susceptibility. Recent studies have highlighted that epigenetic mechanisms may regulate the expression of OA-associated genes. This review aims to present the mechanisms of OA pathogenesis and summarise current evidence regarding the role of genetics and epigenetics in this process.

## 1. Introduction

Osteoarthritis (OA) is the most frequent human joint disease with more than 500 million cases identified in 2019 [1]. Aging, obesity and injuries contribute to the increasing prevalence of the disease [2]. OA is characterised by a progressive degradation of articular cartilage (AC) which is accompanied by damage to the bone, synovium and ligaments [3]. The pathogenesis of OA is heterogeneous and involves metabolic, mechanical and inflammatory factors. An imbalance between the destruction and repair processes of joint tissues leads to a structural impairment [2]. OA may develop in any synovial joint, but is most frequent in the hands, knees and hips [4]. Symptoms of OA include stiffness, pain and the restriction of mobility. OA in the hand also manifests as swellings of the affected joints known as Bouchard and Heberden nodes [5]. Besides the typical OA risk factors (older age, obesity, mechanical load, etc.), recent studies focus on searching for genetic predisposition [6,7]. For instance, Boer et al. identified 100 independent single-nucleotide variants associated with OA [8]. Furthermore, gene expression is further regulated by epigenetic modifications, such as DNA methylation, histone modification or non-coding RNA (ncRNA) interactions. These regulatory mechanisms are also suggested to play a role in OA development. The aim of this review is to present the role of genetics and epigenetics in the pathogenesis of OA.

## 2. Pathogenesis of Osteoarthritis

**Figure 1:**
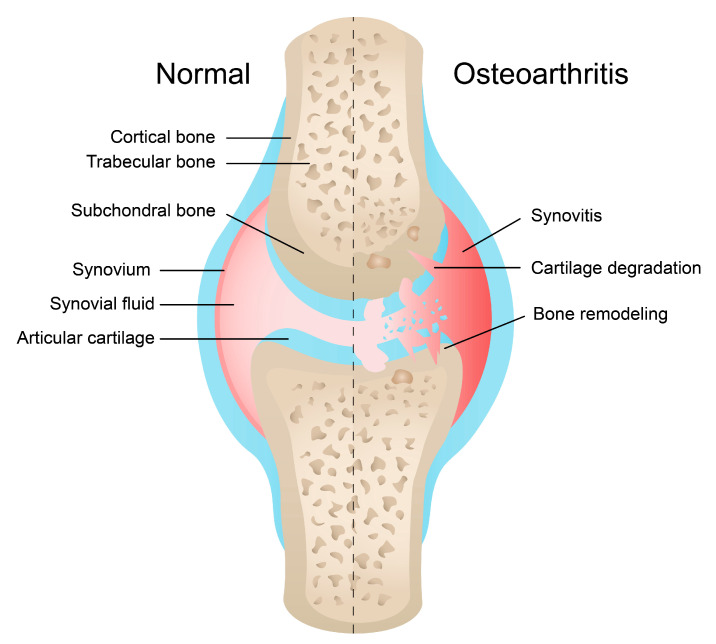
Schematic illustration of healthy and OA joints. OA is characterised by a loss of cartilage, subchondral bone remodelling and inflammation of the synovium.

OA is a whole-joint disease with a multifactorial and complex pathogenesis. Firstly, it is now considered that mechanical injury stimulates inflammatory pathways that are responsible for inducing proteases, which degrade the extracellular matrix (ECM) and contribute to cartilage degeneration [9]. These proteases, together with numerous other cytokines, extracellular-matrix (ECM) proteins, growth factors, and other enzymes, belong to the secretome of chondrocytes [10]. These cartilage-specific cells orchestrate ECM remodelling which can be a physiological or pathological process. The joint is surrounded by the synovium, a fibrous capsule which produces the synovial fluid. Inflammation of the synovium is one of the hallmarks of OA (Figure 1). Multiple cells have been identified in this tissue, including fibroblast-like synoviocytes (FLSs), synovial macrophages, neutrophils, mast cells, and endothelial cells, among others. In a pathological condition, these cells create a pro-inflammatory environment with the presence of proteolytic enzymes, which facilitates joint destruction [11]. Interestingly, Sohn et al. identified 108 proteins in synovial fluid from OA patients. Multiple molecules were associated with inflammation, a process which is thought to originate in the joints in the case of OA. Indeed, the authors observed elevated levels of several molecules in synovial fluid rather than in serum, such as interleukin-6 (IL-6), vascular endothelial growth factor (VEGF), or macrophage chemotactic protein 1 (MCP-1), among others [12].

Protease-induced cartilage destruction is a source of damage-associated molecular patterns (DAMPs). DAMPs induce catabolic or inflammatory mechanisms through pattern-recognition receptors (PRR), such as Toll-like receptors (TLR). For instance, basic calcium phosphate (BCP) crystals, a specific marker of OA, stimulate pro-inflammatory M1 macrophage polarisation [13]. BCP is a common term for calcium phosphate crystals, such as tricalcium phosphate and hydroxyapatite. BCP crystals were also found to promote mitogenesis and stimulate the synthesis of matrix metalloproteinases (MMPs) and prostaglandins, acting as growth factors. Therefore, BCP molecules can activate chondrocytes and synoviocytes through various signalling pathways [14]. High mobility group box 1 protein (HMGB1) and S100A8/A9 (calprotectin) are other DAMPs which can induce inflammatory mechanisms when released from dying or injured cartilage cells [15]. Moreover, released molecules induce inflammatory changes in the synovium which are characterised by stromal vascularisation, hyperplasia and fibrosis [16]. Chwastek et al. showed that stimulated synoviocytes undergo molecular changes and secrete more chemokines and growth factors than healthy cells. Therefore, synoviocytes play a major role in OA pathogenesis and in the altered microenvironment of the joints [17].

Another important hallmark of OA is bone remodelling. It is characterised by microfractures and neovascularisation, which is followed by the migration of immune cells into fracture sites. In OA, both osteoblasts and osteoclasts are stimulated. Nevertheless, osteoclasts cause perforations which breach into the deep layers of the cartilage. Subsequently, cells and fluids can move between the joint cavity and the bone [18]. Subchondral bone changes depend on the stage of OA. Early stages are associated with a thinner subchondral bone and increased trabecular separation. As the disease progresses, subchondral bone sclerosis develops, which is associated with thicker layers and decreased trabecular separation. Furthermore, subchondral cysts and bone marrow edema-like lesions develop [19].

Interestingly, recent studies started to uncover the potential role of a dysregulated microbiome in the progression of OA. The abundance of gut microbiome (bacteriome, mycobiome and virome) is significantly altered in OA patients compared to healthy controls. For instance, higher levels of *Actinobacteriota* and *Proteobacteria* are observed in OA patients [20]. Huang et al. investigated the role of the gut microbiome on OA progression in meniscal/ligamentous injury in mice models. The authors observed significantly greater cartilage damage in animals after fecal microbiome transplantation from patients with OA and metabolic syndrome. Moreover, the same group was associated with higher levels of pro-inflammatory cytokines in the plasma [21]. Recently, an abundance of *Streptococcus* species has been significantly associated with OA knee pain [22]. Therefore, the pathogenesis of OA is a complex process that may include several mechanisms, such as injuries, inflammation, and a dysregulated microbiome. Multiple cells take part in the articular cartilage degradation and may form a positive feedback loop, thus contributing to the pathogenesis of OA.

## 3. Molecular Landscape of Osteoarthritis and Genetic Polymorphisms

### 3.1. Pattern Recognition Receptors

**Figure 2:**
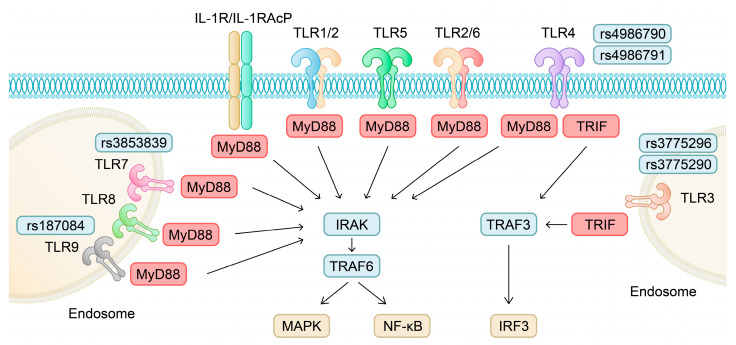
Schematic illustration of the TLR signalling pathways with highlighted gene polymorphisms involved in OA susceptibility. IRAK—interleukin-1 receptor-associated kinase; MyD88—myeloid differentiation primary response 88; TLR—Toll-like receptor; TRAF–TNF receptor-associated factor; TRIF–TIR-domain-containing adaptor-inducing interferon B.

Pattern recognition receptors belong to the innate immune system and respond to microbial elements not related to the host, as well as host molecules which appear due to tissue damage. These molecules are known as pathogen-associated molecular patterns (PAMPs) and damage-associated molecular patterns (DAMPs), respectively. TLR, NOD-like receptors (NLRs) and RIG-I-like receptors (RLRs) are some of the known PRRs [23]. TLRs are transmembrane proteins and their interactions with DAMPs result in the activation of the expression of various genes, such as interleukin 1 (IL-1), TNFα and intercellular adhesion molecule-1 (ICAM-1), among others. The presence and upregulation of all human TLR members (TLR1-TLR10) has been observed in articular cartilage in OA [24]. TLR10 is the only member of the family with anti-inflammatory properties [25]. TLRs are differently distributed in cells: TLR1, TLR2, TLR4, TLR5, TLR6 and TLR10 are expressed on the cell membrane, while TLR3, TLR7, TLR8 and TLR9 are found in the endosomes [26]. Except for TLR3, one of the main downstream adaptor proteins for the rest of the TLR family is myeloid differentiation primary response 88 (MyD88) [27]. Subsequently, the signalling pathway recruits the family of serine–threonine kinases, including interleukin-1 receptor-associated kinase (IRAK) [28]. IRAK members can activate TNF receptor-associated factor 6 (TRAF6), which is a ubiquitin ligase. TRAF6 then stimulates transformation growth factor beta-activated kinase 1 (TAK1), which is followed by the activation of mitogen-activated protein kinase (MAPK) and the transcription factor nuclear factor kappa B (NF-κB). Downstream elements of TLR4 are both MyD88 and TIR-domain-containing adaptor-inducing interferon B (TRIF). TRIF interacts with TRAF6 and TNF receptor-associated factor 3 (TRAF3). The stimulation of TRAF3 leads to the activation of interferon regulatory factor 3 (IRF3). Thus, TLR4 promotes the synthesis of interferon (Figure 2) [29]. TLR3 is the only member of the TLR family whose signalling pathway is MyD88-independent [30]. Several studies have confirmed that members of the TLR family contribute to the pathogenesis of OA and OA-related pain [31,32,33,34].

Furthermore, many studies have evaluated genetic predisposition for OA among TLR-encoding genes. The *TLR9* gene polymorphism rs187084, located in the promoter region, has been associated with a greater risk of OA in the knee (CC genotype) [35,36] and hip (A allele) [37]. The authors identified different alleles which increase the risk of OA. The results may come from different populations or disease phenotypes of the included patients. Therefore, the rs187084 polymorphism may promote transcription and increase the expression of TLR9 in OA. However, Su and colleagues demonstrated that the -1486TT genotype was associated with a higher risk for the development of the disease (with the CC genotype as a reference) [38]. A statistically significant genotype distribution has been identified in the *TLR7* rs3853839 polymorphism as well (genotype GG was more common in patients). Interestingly, the authors observed that the GG genotype occurred in 13% of controls and 28% of patients [39]. According to the 1000 Genome Project, important allele differences between populations could be observed. Frequencies of alleles C and G in European and East Asian populations were 0.83 and 0.17 vs. 0.22 and 0.78, respectively [40]. In SLE patients, carriers of the G allele were shown to have higher TLR7 expression [41]. Yang et al. showed that two *TLR3* polymorphisms—rs3775296 and rs3775290—are associated with an increased risk of OA. The authors also evaluated rs5743312, which showed no associations with the disease. However, after combing the results from this two-stage study, allele C of rs3775291 was associated with reduced risk (OR 0.85; 95% CI 0.72–0.99). Interestingly, chondrocytes with various genotypes of the promoter SNP rs3775296 responded differently to dexamethasone treatment, which indicates that different TLR3 expression rates may induce a higher OA risk. Chondrocytes with the CC genotype, which was associated with the OA risk, also showed higher mRNA *TLR3* expression. In this study, the distribution of alleles A (rs3775296) and T (rs3775290) ranged from 0.21 to 0.28 and from 0.36 to 0.38, respectively [42]. According to the 1000 Genome Project, overall and European MAFs of the rs3775296 are 0.18 and 0.17, respectively. Both global and European MAFs of the rs3775290 are approximately 0.27 and this value tends to be higher in Asian populations (around 0.39 in a Japanese population) [40]. Genotype distributions of rs4986790 and rs4986791 *TLR4* polymorphisms were also significantly different between controls and patients (the respective genotypes AG and CG were more frequent in OA). These polymorphisms are associated with increased OA susceptibility according to the study by Stefik et al. [39]. In addition, the polymorphisms in *TLR10* are also linked with OA predisposition. Vrgoc et al. demonstrated that AA homozygotes of rs11096957 have an approximately 40% higher risk of hip OA. However, the authors did not find any significant differences in allele frequency and knee OA [43]. A separate study by Tang et al. confirmed that among 14 *TLR* polymorphisms, rs11096957 is associated with the development of hip OA. TLR10 is the only member of the TLR family with anti-inflammatory properties. Since rs11096957 is a missense polymorphism, it may alter the structure and function of the receptor, which would explain the increased risk of OA [44]. The receptor for advanced glycation end products (RAGE) is another member of the PRR family. It is a transmembrane protein, which takes part in cell signalling, inflammatory responses and the generation of reactive oxygen species (ROS), among others. Ligands of RAGE include HMGB1, amyloid-B, or S-100 calcium-binding protein [45]. Cecil et al. found that RAGE contributes to OA pathogenesis by promoting inflammation and chondrocyte hypertrophy [46]. The S allele and SS genotype of the rs2070600 polymorphism were found to be more prevalent in the knee OA Chinese population. Moreover, the risk of OA development further increases in SS carriers with obesity [47].

### 3.2. Interleukin 1

IL-1 is a family of cytokines involved in complex inflammatory pathways, which have been correlated with the pathogenesis of numerous diseases. The family consists of 11 members: IL-1α, IL-1β, IL-1Ra, IL-18, IL-33, IL-36Ra, IL-36α, IL-36β, IL-36γ, IL-37 and IL-38. Most of the members exhibit proinflammatory properties. On the other hand, IL-37 is an anti-inflammatory mediator, while the precise role of IL-38 remains unknown and it has been found to both inhibit and stimulate inflammatory responses [48,49]. Moreover, IL-1, together with DAMPs, further stimulates the expression of TLRs [50]. Members of the IL-1 family modulate a cellular context through different receptors (IL-1R). These receptors have a cytoplasmic domain known as a Toll interleukin-1 receptor (TIR), which is homologous to the domains in TLRs. Furthermore, the signalling pathway of IL-1 also involves MyD88, NF-κB and MAPK stimulation [51,52]. Therefore, downstream effectors of IL-1 also promote pro-inflammatory conditions and cartilage degradation. A study by Goekoop et al. demonstrated that IL-1ß takes part in OA pathogenesis. The authors revealed that the low innate production of IL-1ß is a protective factor for OA in old age [53]. Furthermore, higher innate ex vivo IL-1ß production upon lipopolysaccharide (LPS) stimulation is associated with greater risk of OA [54]. However, investigations using IL-1-deficient collagenase-induced OA mice showed that the lack of IL-1 was not associated with significant reductions in mRNA expression of proteases. However, IL-6 expression was statistically reduced in the IL-1-deficient model compared to wild controls 7 days after the induction of OA [55]. IL-1 signalling was found to promote the expression of MMP-2, MMP-8, MMP-9, MMP-12 and MMP-13 in chondrocytes [56,57,58]. Furthermore, Lee et al. demonstrated that IL-1α promotes the expression of Piezo1, an ion channel in chondrocytes. As a result, increased Ca^2+^ intracellular flow occurs, which increases remodelling and damage to the cartilage [59]. In addition, IL-1ß induces inflammatory responses in synoviocytes. IL-1ß synergises with HMGB1 to release the proinflammatory cytokines IL-6 and IL-8, the chemokines CCL2 and CCL20, and MMPs [60]. Nevertheless, IL-1ß is usually detected at very low concentrations in OA, and its direct impact on disease progression is difficult to establish. However, Gruber et al. showed that the explantation of animal cartilage and applying mechanical injury stimulates IL-1ß mRNA expression [61]. In clinical trials, the use of agents targeting IL-1 showed limited benefits, which may indicate that IL-1 is not the key cytokine driving the progression of OA [62,63,64].

Genes encoding proteins of the IL-1 family are located on chromosomes 2, 9 and 11. Corresponding genes for the receptors IL-1R1 and IL-1R2, as well as cytokines IL-1α and IL-1ß, are found in a cluster in chromosome 2 [65]. However, the literature describes conflicting results regarding polymorphisms of the IL-1 family of genes and the risk of OA. Kaarvatn et al. showed that there were no allele differences in the distribution of *IL-1A* SNP rs1800587 and *IL-1ß* SNP rs1143634 between the knee and hip OA patients and controls. Nevertheless, in the former SNP, the C/T genotype was associated with an elevated risk of knee OA (OR 1.39; 95% CI 1.01–1.92) [66]. The next two polymorphisms are located in the promotor region of the *IL-1ß* gene: rs16944 (−511C>T) and rs1143627 (−31T>C). The occurrence of the T and C alleles in the respective variants is associated with the increased secretion of IL-1ß [67]. Furthermore, studies involving patients with different diseases showed that TT genotype of rs16944 was associated with elevated IL-1ß levels [68,69]. However, Ni et al. found no significant differences in the frequency of rs16944 in healthy cases and OA patients [70]. Additionally, the −511G>A polymorphism also showed no predisposition towards OA in the Croatian population [71]. A large meta-analysis performed by Kerkhof and colleagues showed no association between the three, rs1143634, rs16944, and rs419598, with OA [72]. However, Stern et al. demonstrated that *IL-1* gene polymorphisms may contribute to an erosive hand OA [73]. The IL-1R antagonist (IL-1Ra) binds to the IL-1R1 and inhibits pro-inflammatory IL-1 signalling [74]. It can suppress the catabolic state of the chondrocytes induced by IL-1 [75]. A meta-analysis performed by Budhiparama et al. indicated that the IL-1RN*1 allele decreases the risk of a knee OA (OR 0.67; 95% CI 0.48–0.95), while IL-1RN*2 elevates the risk (OR 1.38; 95% CI 1.02–1.85) [76]. Moreover, Wu and colleagues found that seven *IL1RN* polymorphisms were associated with the progression of the disease (OR was significantly positive in five SNPs). Furthermore, a haplotype of rs419598, rs9005 and rs315943 was also associated with radiological progression of knee OA [77,78].

Furthermore, in synoviocytes obtained from OA patients, IL-18 (another member of the IL-1 family) promotes synovitis by enhancing the expression of TNFα, prostaglandin E2 (PGE2) and cyclooxygenase 2 (COX2) [79]. Koh and colleagues revealed that significantly higher concentrations of IL-18 are detected in the synovial fluids of OA knees [80]. The wild-type allele C of the IL-18 promoter SNP rs1946518 is found more often in knee OA than in controls. Furthermore, the haplotype composed of alleles CG of the respective rs1946518 and rs187238 variants was found to be a potential risk factor for OA [81]. Interestingly, rs1946518 SNP may be associated with transcriptional activity, as AA homozygotes showed reduced IL-18 mRNA expression in stimulated peripheral blood mononuclear cells [82].

### 3.3. Interleukin 6

**Figure 3:**
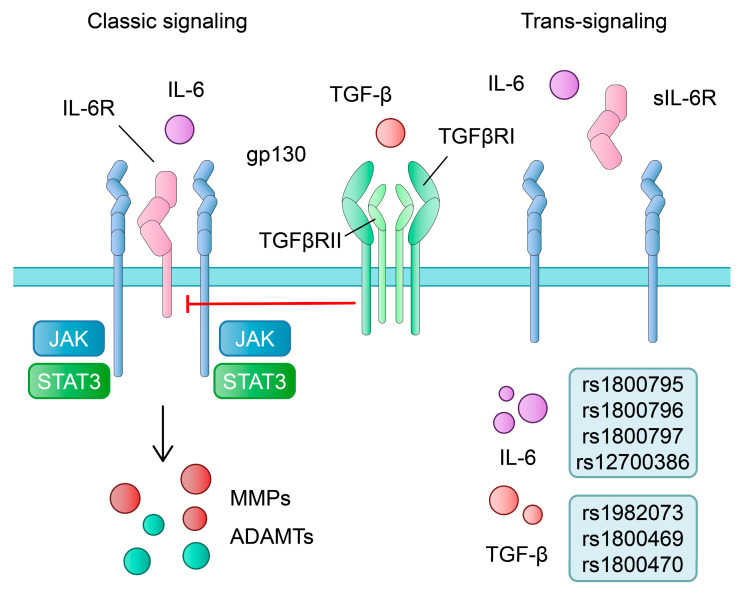
Schematic and simplified IL-6 signalling pathway, its impact on OA-related metalloproteinases and selected polymorphisms associated with OA development or protection. ADAMTS—a disintegrin and metalloproteinase with thrombospondin motifs; IL-6—Interleukin-6; JAK—Janus kinase; MMP—matrix metalloproteinase; STAT3—signal transducer and activator of transcription 3; TGF-β—transforming growth factor β.

Interleukin-6 (IL-6) is one of the most significant pro-inflammatory cytokines. It is secreted upon TLR stimulation in myeloid cells [83]. The IL-6 signalling pathway has been correlated with the development of inflammatory diseases, such as rheumatoid arthritis [84], COVID-19 infection [85] and atherosclerosis [86], among others. The classic signalling pathway involves IL-6 binding to its receptor, IL-6R, which is bound to the cellular membrane. Subsequently, after interaction with the subunit gp130, downstream signalling stimulates the Janus kinase (JAK) and the signal transducer and activator of transcription 3 (STAT3) (Figure 3). This pathway usually operates in immune cells and hepatocytes. The trans-signalling pathway involves soluble IL-6R (sIL-6R), which binds to IL-6 in the circulation. The newly formed complex signals through gp130 are expressed on various cell subtypes [87]. The IL-6 signalling pathway is also associated with bone remodelling, as the activity of both osteoblasts and osteoclasts seems to be regulated by this cytokine. Furthermore, chondrocytes also express IL-6 and IL-6R [88].

Recent evidence suggests that IL-6 plays a significant role in the pathophysiology of OA. To begin with, de Hooge et al. demonstrated that IL-6 knockdown promoted age-related OA in male mice [89]. In contrast, Liao and colleagues demonstrated that the ablation of *IL6* results in the inhibition of injury-induced cartilage catabolism in mice. Furthermore, the authors showed that gene deletion suppressed post-traumatic OA pain responses [90]. Additionally, IL-6 promotes cartilage catabolism and degradation. Latourte et al. showed that IL-6 treatment of mouse chondrocytes induces the expression of MMP-3 and MMP-13, together with a disintegrin and metalloproteinase with thrombospondin motifs (ADAMTS) 4 and ADAMTS 5 [91]. A similar association between IL-6 and MMPs was also found in a study by Ryu et al. [92]. Moreover, the treatment of mouse chondrocytes with IL-6 promotes the secretion of MCP-1, CCL5 and CXCL12. The inhibition of JAK2/STAT3 reduces the secretion of pro-inflammatory cytokines and suppresses cartilage degeneration in vivo [93]. Moreover, the neutralisation of IL-6 downregulates *MMP13* gene expression in synoviocytes and chondrocytes [94]. Interestingly, Liang and colleagues demonstrated that the IL-6/STAT3 pathway might have an additional upstream regulator in OA-a retinoic acid receptor-related orphan receptor-α (RORα). The authors showed that the expression of RORα is elevated in the OA AC of patients and mice. Furthermore, the inhibition of RORα inhibits the IL-6/STAT3 pathway and attenuates cartilage damage [95]. Choi et al. suggested that the development of OA is correlated with alterations of cholesterol, while RORα links these two processes [96].

Several factors promote the secretion of IL-6 in OA-related cells. For instance, obesity enhances the secretion of IL-6 from synovial fibroblasts [97]. Furthermore, particulate matters (PM) with a diameter of less than 2.5 μm were found to promote the production of IL-6 in OA synovial fibroblasts through the generation of reactive oxygen species (ROS) [98]. In addition, insulin promotes IL-6 expression through the nuclear factor kappa B (NF-ĸB) in fibroblast-like synoviocytes [99]. Intriguingly, Wiegertjes and colleagues showed that transforming growth factor-β (TGF-β) promotes IL-6 expression, but downregulates IL-6R, which suppresses IL-6-signalling in chondrocytes [100]. IL-6 might be in a positive feedback loop with BCP. A study by Nasi et al. revealed that the exposure of murine chondrocytes to BCP increased the secretion of IL-6. Additionally, IL-6 is capable of increasing the formation of BCP crystals [101].

Several studies evaluated the potential role of *IL-6* genetic variants and susceptibility to OA. IL-6 is located on the short arm of chromosome 7 [102]. To begin with, it seems that the distribution of rs1800795 alleles greatly differs between various populations. According to the 1000 Genome Project, their overall MAF is 0.14. However, in the European population, frequencies of alleles C and G are 0.42 and 0.58, respectively [40]. Conflicting results have been published regarding the rs1800795 (-174 G/C) SNP in OA. Singh et al. demonstrated that the MAF of rs1800795 was significantly decreased in patients with OA. Major allele G was correlated with an increased risk of the disease. Furthermore, the GG genotype was associated with increased plasma levels of IL-6 and IL-1ß [103]. However, a recent meta-analysis showed that there were no associations between rs1800795 and OA [104]. Nevertheless, a recent study found that the G allele and GG genotype were more frequent in OA cases in the Turkish population [105]. In contrast, the C allele of rs1800795 was associated with an increased risk of knee OA in a study by Sun et al. [106]. Surprisingly, the CC genotype of the same SNP was correlated with a higher expression of IL-6 in synovial fibroblasts [107]. Interestingly, the role of the rs1800795 polymorphism seems to be more complex. Białecka et al. demonstrated that patients with the G allele and GG genotype undergoing total hip replacement required more opioids than those with the CC genotype [108]. The rs1800796 *IL-6* gene polymorphism is composed of the alleles C and G. Allele G and genotype GG were more common in the cohort with OA. The presence of allele C was associated with reduced susceptibility to OA [109]. However, Yigit and colleagues did not find differences between patients and controls regarding -572 G/C variant [105]. A meta-analysis by Deng et al. showed that the G allele and GG genotype increase the risk of OA [104]. Stingh et al. showed that MAF of rs1800796 was significantly reduced in the OA cohort, while allele G showed elevated risk for the disease in a recessive model. Moreover, higher plasma levels of IL-6 and IL-1ß were detected in GG canotype carriers [103]. The presence of the G allele of SNP rs1800797 was more frequent in patients with symptomatic OA of the distal interphalangeal joints [110]. Furthermore, the C allele and CC genotype of the rs12700386 SNP increases the risk of a knee OA in the Chinese Han population [111]. The *TGF-β1* SNP rs1982073 was also found to increase the risk of knee OA (allele C; genotypes CT and CC) [112]. TT genotype (rs1800470), as well as T allele and TT genotype (rs1800469) were correlated with the occurrence of OA [113]. In addition, carriers of selected *TGF-β1* gene variants may have antagonistic or synergistic interactions with obesity, increasing the risk of OA [114].

### 3.4. Matrix Metalloproteinases

Matrix metalloproteinases are zinc-dependent proteases which play a key role in ECM degradation. Their presence was observed in multiple types of cells. However, due to their role in ECM remodelling, MMPs are abundantly distributed in the connective tissues [115]. MMPs take part in degrading aggrecan, which is a typical hallmark of OA [116]. The family comprises 23 enzymes in humans [117] and their increased levels were identified in both the blood and SF of OA patients [118,119,120,121]. Based on their structure and substrate specificity, the family is divided into several subgroups, including typical proteinases (e.g., MMP-1,-9,-13), gelatinases (MMP-2, MMP-9), matrilysins (MMP-7, -16) and those that are membrane bound (e.g., MMP-14, -15, -17), among others [122]. These molecules are secreted by neutrophils [123], macrophages [124], chondrocytes [125] and synoviocytes [17]. Intercellular interactions contribute to the progression of OA. For instance, the conditioned medium of activated macrophages upregulates MMP-3 and MMP-13 in chondrocytes [126]. Major attention has been paid to the role of MMP-13, also known as collagenase-3, in the pathogenesis of OA. It degrades ECM components, together with collagen types I, II and III [127]. Elevated serum levels of MMP-13 are correlated with structural impairment of the knee in OA patients [128]. The upregulation of MMP-13 occurs through various signalling pathways, including PI3K/Akt/NF-κB and MAPK [129,130]. In an OA mice model, Little and colleagues demonstrated that MMP-13 knockdown results in the suppression of cartilage damage [131]. Due to the major role of MMP-13 in OA progression, much effort has been placed on the development of selective inhibitors [132,133,134].

Overall, MMPs play a significant role in cartilage degeneration and the progression of OA. Therefore, genetic variations in MMP-encoding genes have been extensively studied. To begin with, the SNP in the promoter region of *mmp1* (-1607 1G/2G, rs1799750) has been suggested to play a role in the development of OA. Due to inconsistent data in the literature, Liu et al. performed a meta-analysis, which showed that the rs1799750 polymorphism might be related to susceptibility of the temporomandibular joint to OA and to the disease in younger populations [135]. However, no association between rs1799750 and knee OA was found in a recent study by Kao and colleagues [136]. Guo et al. revealed that four SNPs in the *MMP3* genes were associated with an increased risk of OA: rs639752, rs520540, rs602128 and rs679620 [137]. In addition, allele A of the *MMP13* rs2252070 polymorphism was more common in the cohort of patients with knee OA [106]. In vitro experiments showed that allele A was associated with the elevated transcriptional activity of MMP-13 [138].

### 3.5. A Disintegrin and Metalloprotease with Thrombospondin Type I Motifs (ADAMTS)

ADAMTS, a family of extracellular proteases found both in invertebrates and mammals, is associated with several diseases such as cardiovascular disease, dysgenesis, cancer and arthritis. In the structure of ADAMTS, several domains can be distinguished: a signal sequence, a prodomain, protease and disintegrin domains, a thrombospondin type 1 motif, a cysteine-rich region, and a spacer domain [139]. To date, 19 members of this family and 7 ADAMTS-like proteins have been identified and divided into five groups based on their function. The first one, so-called aggrecanases, includes ADAMTS 1, 4, 5, 8, 9, 15 and 20. The subsequent group, known as procollagen N-peptidases, comprises ADAMTS 2, 3 and 14. ADAMTS 7, and 12 belong to the cartilage oligomeric matrix proteolytic enzymes group. In turn, ADAMTS 13 is a von-Willebrand factor proteolytic enzyme. ADAMTS 6, 10, 16, 17, 18 and 19 were assigned to the last group, termed orphan enzymes [140].

Glasson et al. studied ADAMTS 4 knockout (KO) mice and observed no impact on OA progression as well as no abnormalities of skeletal development, remodelling and growth [141]. In contrast, in another study, siRNA silencing of *ADAMTS 4* and *ADAMTS 5* delayed the degeneration of cartilage tissue. The experimentally induced model of degenerated cartilage demonstrated enhanced *ADAMTS 4* mRNA expression [142]. Moreover, IL-1 promotes ADAMTS 5, while syndecans (proteoglycans upregulated in degenerating cartilage of an OA mice model) are engaged in ADAMTS 4 and ADAMTS 5 activation [143,144]. Furthermore, the expression of ADAMTS 4, 5, 7 and 12 increases with the progress of OA [145]. Therefore, methods for ADAMTS inhibition have been previously investigated [146,147]. Interestingly, while ADAMTS 4 and 5 constitute a subgroup of destructive aggrecanases in OA, ADAMTS 9 may be involved in skeletal development [148,149]. Taking into account that ADAMTS 9 contains a long cytosine–adenine (CA) microsatellite repeat sequence within a promoter region, it was noticed that gene expression levels may be related to variation in the length of these sequences. Thus, CA repeats may constitute a marker of some diseases. In an analysis by Gok et al., the authors found that CA repeat length ≥ 20 was associated with severe radiological knee OA [150]. Moreover, elevated levels of ADAMTS 7 and ADAMTS 12 have been observed in OA as well [151,152].

Ma et al. investigated *ADAMTS 14* gene polymorphism and vulnerability to knee OA in the Chinese Han population. A non-synonymous SNP (nsSNP) of the *ADAMTS 14* gene, rs4747096, is located on chromosome 10q22.1. Analyses of both male and female controls and OA patients identified three genotypes: AA, AG and GG. The minor allele G was detected significantly more often in patients with OA. Allele G and genotype GG were associated with increased risk of the disease [153]. Furthermore, the frequency of the G allele in the ADAMTS 14 gene nsSNP rs4747096 in Caucasian women requiring total knee replacement and patients with symptomatic hand OA was significantly increased in comparison to the controls [154]. Different results were observed by Poonpet et al. [155]. However, Rodriguez-Lopez and colleagues demonstrated that two SNPs of ADAMTS 5 (rs2830585 and rs226794) are probably not related to OS susceptibility [156].

### 3.6. Other Polymorphisms and Genetic Variants

Previous fragments broadly discussed the functions and genetic variants of major receptors, cytokines and enzymes linked with OA. Nevertheless, genetic predisposition has been evaluated in a number of different genes. For instance, rs2279115 (BCL-2, allele C) and rs2277680 (CXCL 16, allele G) were associated with OA [157]. Furthermore, Wang et al. showed that polymorphisms of ITLN1, XCL2 and DOT1L are also linked with OA [158]. ITLN1 or omentin has been recently shown to decrease the expression of MMPs in IL-1β-stimulated chondrocytes [159]. Furthermore, DOT1L is also considered a protective factor in OA through interactions with sirtuin-1 and the Wnt pathway [160,161]. In addition, a matrix Gla protein (MGP) polymorphism rs1800802 was also correlated with OA. According to a study by Hui and colleagues, the GG genotype occurred in 8.2% of OA patients and was associated with susceptibility to OA [162]. Table 1 presents the list of selected gene polymorphisms associated with OA. Interestingly, a mitochondrial activity seems to be disrupted in OA cells. Disease-related cybrids (fusions of platelets with nuclear donor cells) showed higher mitochondrial DNA (mtDNA) copy numbers, which could be a compensatory mechanism, and they act similarly to OA chondrocytes [163]. Moreover, mtDNA might help in identifying patients prone to a progression of OA. Duran-Sotuela et al. demonstrated that mtDNA variant m.16519C is significantly more common in patients with a rapid progression of knee OA. Furthermore, the authors demonstrated that its presence is associated with mitochondrial reactive oxygen species and a different transcriptome regarding IL-6 [164]. Previous studies also showed that mitochondrial haplogroups (sets of mtDNA SNPs) might be associated with disease prevalence, features, or progression [165,166].

## 4. Epigenetic Regulation in Osteoarthritis

### 4.1. DNA Methylation

**Figure 4:**
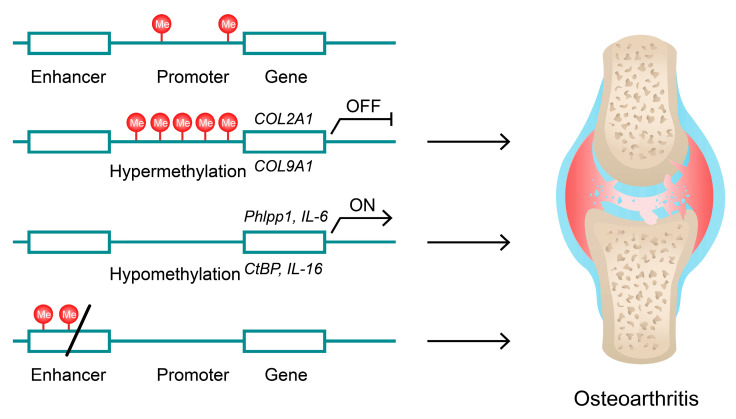
DNA methylation promotes or suppresses transcription of OA-related genes. Stimulated or primary hypermethylation of genes encoding collagen molecules inhibits transcription and promotes OA [180,181]. In contrast, hypomethylation of promoters of pro-inflammatory mediators results in their elevated expression [174,175,176,177]. Furthermore, recent studies showed multiple differently methylated enhancer regions between OA patients and controls [183].

Epigenetic modifications involve processes associated with the regulation of gene expression, such as DNA methylation, histone modifications or chromatin remodelling [167]. Furthermore, RNA epigenetics also largely contributes to the regulation of gene expression [168]. To begin with, methylation of CpG islands is considered a major epigenetic regulation and is involved in processes such as aging and carcinogenesis [169,170]. Depending on the region, CpG methylation may either promote or repress gene expression [171]. Recent studies revealed that DNA methylation is one of the factors contributing to the pathogenesis of OA and its pro-inflammatory character. Firstly, comparison of the OA animal model with controls reveals differences in the methylation rates of selected genes [172]. Furthermore, C-terminal-binding proteins (CtBP1 and CtBP2) act as corepressors of transcription, the levels of which are elevated in patients with OA [173,174]. Sun and colleagues demonstrated that methylation of the CpG island in the promoters of *CtBP* genes was decreased in OA patients, thus apparently being associated with elevated CtBP proteins and their subsequent signalling [174]. Furthermore, hypomethylation of *IL-16* in the OA cohort has been identified, which led to its elevated concentrations in serum. As a result, IL-16 can promote the production of pro-inflammatory mediators, such as IL-6 or TNFα [175]. Protein phosphatase Phlpp1 represents another enzyme that is elevated in OA tissues and has decreased CpG promoter methylation [176]. Similarly, the promoter region of IL-6, which is enriched in CpG sites, is hypomethylated in SF from OA patients. Accordingly, these regions are also associated with decreased binding of Dnmt1 and Dnmt3a [177]. Interestingly, a recent study demonstrated that the depletion of STAT3 in foetal chondrocytes results in the DNA hypermethylation of genes related to aging, proliferation, or development of the ECM, among others. The authors suggest that STAT3 might act through the DNA methyltransferase 3 beta (DNMT3B). The knockdown of STAT3 was associated with progressed OA in post-traumatic animal models [178]. Additionally, Izda and colleagues showed that aging and OA in murine models generated through destabilisation of the medial meniscus share some similarities in DNA methylation, which shows that aging might contribute to the development of OA on an epigenetic level [179]. Zhang et al. revealed that human chondrocytes stimulated with triclocarban showed elevated methylation in the *COL2A1* gene (type II collagen), which suppressed its expression and reduced cartilage tissue [180]. Moreover, *COL9A1* (collagen type IX) promoter is hypermethylated, which results in a decrease in respective mRNA molecule levels in OA [181]. Importantly, the evaluation of a DNA methylation pattern might be used to monitor the progression of OA [182]. Moreover, recent studies have found numerous differently methylated CpGs in enhancer regions [183,184]. Interestingly, the analyses of DNA methylation may help in understanding the precise role of genetic susceptibility. Polymorphisms associated with changes in gene expression may change DNA methylation status. For instance, Kehayova et al. revealed an allelic expression imbalance between C and T alleles in heterozygous hip or knee OA patients. Compared with allele T, allele C of rs583641 was associated with an elevated expression of *COLGALT2* and a reduced DNA methylation of gene enhancer [185]. Therefore, hypomethylation of gene promoters encoding pro-inflammatory proteins, as well as hypermethylation of the genes associated with cartilage quiescence and structure may contribute to the pathogenesis of OA (Figure 4).

### 4.2. Histone Modifications

Histone modifications represent another major epigenetic regulator of gene expression. These alterations have been recently proposed to play a role in the pathogenesis of OA. Classical histone modifications include acetylation, phosphorylation and methylation [186]. Acetylation is performed through acetyltransferases and deacetylases. Hyperacetylation is associated with decondensed chromatin, while deacetylation contributes to the compacted form of chromatin, which affects gene expression [187]. Histone deacetylases (HDACs) facilitate the deacetylation of both histones and nonhistone proteins [188]. Several studies have investigated HDACs expression alterations and their different roles in OA. To begin with, the expression of HDAC1 and HDAC2 is elevated in OA chondrocytes. These enzymes suppress COL2A1 and aggrecan, together with COL9A1 (HDAC1) and COL11A1 (HDAC2) [189].

**Figure 5:**
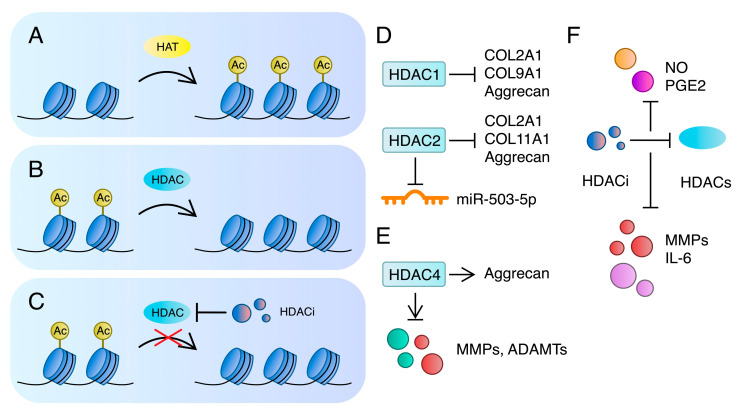
A schematic illustration of (**A**) histone acetylation; (**B**) histone deacetylation; and (**C**) inhibition of histone deacetylases. (**D**,**E**) The role of HDAC2 and HDAC4 in OA-associated catabolic and inflammatory molecules [189,190,192,193,194]. (**F**) HDACis suppress NO, PGE2, MMP, and IL-6, which are associated with OA [196,199,201]. ADAMTS—a disintegrin and metalloprotease with thrombospondin type I motifs; HAT—histone acetyltransferase; HDAC—histone deacetylase; HDACi—histone deacetylase inhibitor; MMP—matrix metalloproteinase; NO—nitric oxide; PGE2—prostaglandin E2.

Studies showed conflicting results regarding the role of HDAC4 in OA. According to Lu et al., it was upregulated in OA cartilage compared to normal tissue. Nevertheless, it might be associated with early OA, since a negative correlation between HDAC4 and OA severity was observed. The authors also showed that the suppression of HDAC4 blocked the expression of MMPs and ADAMTS4 in stimulated chondrocytes. In contrast, *ADAMTS5* mRNA was promoted in an HDAC-silenced experiment. Furthermore, the suppression of HDAC4 promoted aggrecan mRNA expression in stimulated and unstimulated cells, while the impact of HDAC4 silencing on mRNA levels of *COL2A1* was dependent on stimulation status [190]. In contrast, Cao and colleagues demonstrated that the expression of HDAC4 is decreased in OA tissues. Furthermore, the authors showed that HDAC4 promotes type II collagen and inhibits MMPs, type X collagen, and ADAMTS, among others [191]. Therefore, the role of HDAC4 seems to be more complex in OA. In mice, HDAC4 deletion results in a spontaneous OA development, promotion of *MMP-13* mRNA expression and inhibition of type II collagen and aggrecan [192]. Accordingly, the overexpression of HDAC4 promotes type II collagen, aggrecan, and inhibits *MMP-13* and *Col X* mRNA expression [193]. HDACs may also modify the expression of non-coding RNA (ncRNA). For instance, HDAC2 suppresses miR-503-5p, which targets serum- and glucocorticoid-inducible kinase-1 (SGK1). Consequently, HDAC2 promotes SGK1, which contributes to inflammation [194] (Figure 5). Furthermore, HDAC4 interacts with miR-483-5p, as its downregulation promotes the expression of this ncRNA. MiR-483-5p can target *COL2A1* and, consequently, promote OA [195].

Importantly, the inhibition of HDACs has been associated with suppression of the disease. For instance, the use of the HDAC inhibitor (HDACi) resulted in the reduction of the fibroblast growth factor-2 (FGF2)-induced production of MMP-1 and MMP-13 in chondrocytes. However, the inhibitor further decreased COL2A1 and aggrecan in human chondrocytes. It is suggested that the effect of HDACis may depend on the stage of OA [196]. According to Furumatsu et al., trichostatin A increased COL2A1 and aggrecan expression after 4 h of treatment. Nevertheless, a reducing trend was observed at 8 h of treatment in chondrocytes [197]. Similar results were observed in the levels of nitric oxide and prostaglandin E2, which have been correlated with OA [198,199]. Zhong et al. demonstrated that vorinostat, an HDACi, inhibited IL-1β-induced MMP-1 and MMP-13 in chondrocytes [200]. Moreover, a recent study showed that panobinostat (HDACi) suppressed basal and IL-1β-induced inflammatory IL-6, among other OS markers. Furthermore, the authors also showed that panobinostat alleviated OA in mice [201]. Importantly, nuclear factor erythroid 2-related factor 2 (NRF2) is chondroprotective and might suppress cartilage degeneration [202,203]. NRF2 acetylation promotes its signalling; therefore, inhibiting HDAC could promote its protective pathway. Cai and colleagues demonstrated that the use of HDACi in the NRF2-KO mouse model is associated with a minimal reduction of cartilage damage. In contrast, significant improvements were observed in wild-type mice. The authors concluded that the protective role of HDACi in OA is NRF2-dependent [204].

Sirtuins (Sirt) are NAD+-dependent deacetylases, which belong to the family of histone deacetylases. Seven known proteins belong to this family. Sirt1 is considered chondroprotective, as its deletion is associated with OA in mice, while OA patients have reduced levels of Sirt 1 [205]. One of the explanations for reduced Sirt1 in OA is the possible hypermethylation of CpG in the Sirt1 promoter [206].

Another histone-concentrated mechanism, which is possibly involved in OA, is histone methylation. Lysine and arginine residues may be methylated, which can induce contradictory effects. For instance, H3K9 methylation is classically associated with repressed gene expression, while adding methyl groups to H3K4 promotes expression [207]. In chondrocytes, IL-1 treatment resulted in the promotion of iNOS and COX2, which was accompanied by the elevation of H3K4. The suppression of SET1A (methyltransferase) decreased the IL-1-induced expression of iNOS and COX2 [208]. Mansouri et al. showed that human OA chondrocytes treated with IL-1β promoted microsomal prostaglandin E synthase 1 (mPGES-1) mRNA expression and decreased H3K9 mono- and di-methylation in its promoter [209]. Furthermore, the suppression of H3K9 methylation is associated with increased MMP-1 and MMP-13 in mouse chondroprogenitor cells [210].

Enhancer of zeste homolog 2 (EZH2) is a histone methyltransferase, which catalyzes the methylation of H3K27. OA chondrocytes show significant H3K27me3 immunostaining [211]. This modification is usually associated with the repression of transcription. For instance, EZH2 methylates histones in the miR-138 promoter region, which silences its transcription and promotes OA [212]. However, in human chondrocytes stimulated with IL-1, EZH2 further increases mRNA expression and the release of MMPs and IL-6, as well as the production of NO and PGE2. EZH2 inhibition suppresses IL-1β-stimulated chondrocyte inflammation and reduces cartilage degradation in vivo [213]. Interestingly, EZH2 might impact mRNA expression independently of its methyltransferase role, especially considering that the loss of Kdm6a, a H3K27me3 demethylase, was associated with alleviated OA in mice. Furthermore, intraarticular injections of the Kdm6a inhibitor suppressed OA progression [214]. Furthermore, the knockout of Utx, another H3K27 demethylase, promotes the expression of ECM markers and inhibits OA development. Surprisingly, Utx deletion reduces H3K27me3 as well. However, Lian et al. showed that Utx deficiency promoted the levels of EZH2, but decreased Suz12 and Eed, which participate in the histone methylation process [211]. Interestingly, EZH2 might take part in DNA methylation and hydroxymethylation processes, which can impact transcription, as demonstrated in hyperglycaemic conditions in retinal endothelial cells [215]. EZH2 has been shown to promote the methylation of its target’s promoters, which has also been associated with gene silencing [216]. Moreover, in ultraviolet-treated fibroblasts, EZH2 was found to simultaneously promote the expression of MMP-1 and inhibit COL1A2. Transcriptional activation was performed through the interaction with components of NF-κB at the MMP-1 promoter region [217]. Therefore, EZH2 seems to take part in several epigenetic mechanisms associated with gene expression. Further research is required to better understand its role in OA. Moreover, the stimulation of chondrocytes with IL-1β results in the elevated expression of several histone demethylases, such as *KDM2A, KDM6A* and *KDM7A*, among others. The inhibition of KDM2/7 increases the methylation of H3K79 and suppresses cartilage damage in mice [218].

### 4.3. Non-Coding RNA

**Figure 6:**
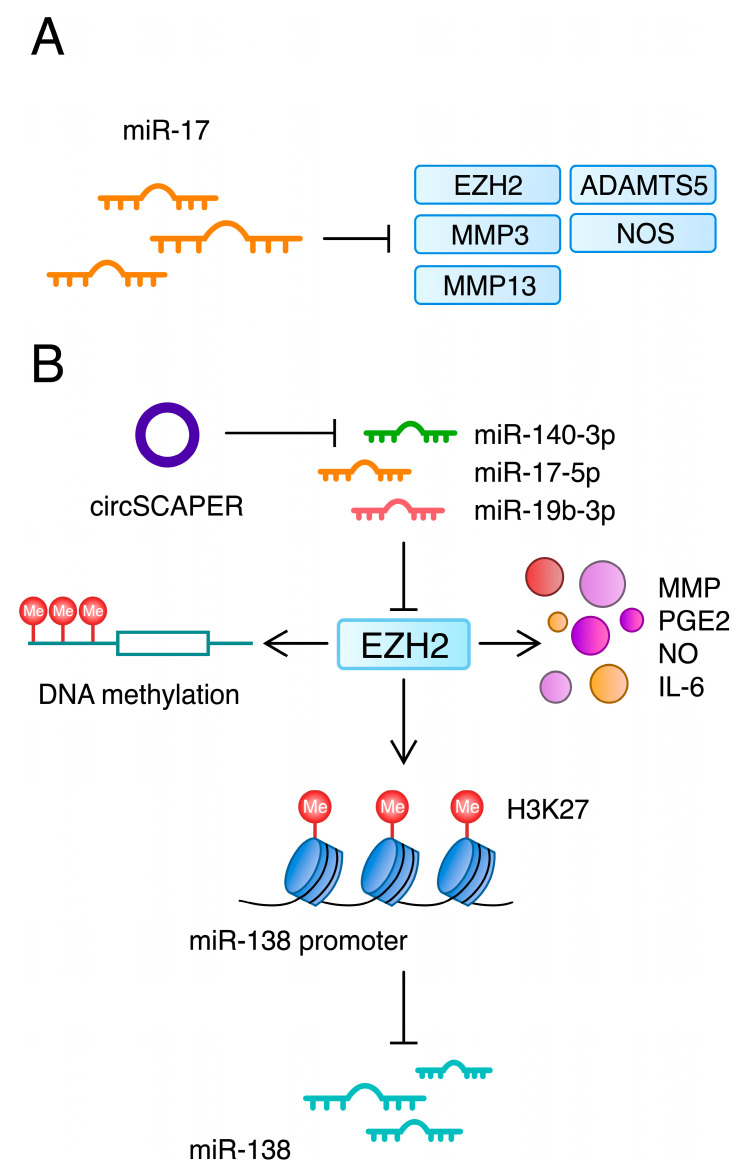
(**A**) A chondroprotective role of miR-17 [225]; (**B**) The various functions and interactions of EZH2, a histone methyltransferase, which methylates H3K27 [212] and promotes OA-related markers and catabolic factors in stimulated chondrocytes [213]. EZH2 is involved into the regulatory network with other epigenetic mechanisms, such as DNA methylation [216], and it is a target of several micro RNAs [229,230]. ADAMTS—a disintegrin and metalloprotease with thrombospondin type I motifs; EZH2—enhancer of zeste homolog 2; MMP—matrix metalloproteinase; NO—nitric oxide; PGE2—prostaglandin E2.

Non-coding RNAs represent a heterogenous group of RNA molecules, which include long non-coding RNAs (lncRNAs), circular RNAs (circRNAs) and small non-coding RNAs (sncRNAs). sncRNAs are further divided into microRNAs (miRNAs), small-interfering RNAs (siRNAs) and piwi-interacting RNAs (piRNAs), among others [219]. These molecules differently alter gene expression. For instance, miRNAs can post-transcriptionally silence gene expression, while lncRNAs and circRNAs may act as endogenous competitor RNAs or sponges [220,221]. Recent studies have highlighted that ncRNAs take part in the progression or suppression of OA. To begin with, miRNAs can be abundantly present in the organism or be specifically expressed in certain tissues. According to a study by Ludwig et al., the majority of miRNAs have an intermediate specificity regarding tissue expression [222]. Recently, miR-3085-3p has been found to be selectively expressed in cartilage, which targets *ITGA5*, *ACAN* and *COL2A1*, indicating its possible role in OA [223,224]. Zhang and colleagues revealed that the expression of miR-17 was decreased in OA mice. The authors found that miR-17 suppressed *Nos2*, *ADAMTS5*, *MMP3* and *MMP13*. The transfection of miR-17 mimic into IL-1β-treated mouse chondrocytes reduced the elevated expression of these factors. Similar results were observed when agomir-17 was applied in vivo [225]. Additionally, a recent study demonstrated that miR-149 is downregulated in OA cartilage. Vascular cell adhesion molecule 1 (VCAM-1) was found to be a direct target of miR-149. The upregulation of miR-149 was associated with suppressed inflammation [226]. Interestingly, miRNAs may also promote the progression of OA. Endisha and colleagues showed that the expression of miR-34a-5p was increased in the synovial fluid, plasma and cartilage of OA patients when compared with controls. Human OA chondrocytes treated with miR-34a-5p mimic showed the decreased expression of *COL2A1* and *ACAN* alongside elevated *MMP13* and *ADAMTS5* [227]. Interestingly, interactions between HDACs and ncRNA also play a role in the development of OA. For instance, miR-222 targets HDAC4 and, therefore, suppresses MMP-13 in chondrocytes [228]. Moreover, ncRNAs interact with histone methyltransferases as well. Li et al. revealed that miR-17-5p and miR-19b-3p target EZH2 and inhibit OA progression [229]. Furthermore, circSCAPER promotes ECM degradation through the binding to miR-140-3p, which also targets EZH2 [230] (Figure 6). CircSCAPER was also found to sponge miR-127-5p and stimulate the expression of TLR4 [231]. In addition, the expression of circTBX5 is elevated in OA cartilage tissues. It targets miR-558, which negatively regulates MyD88. The knockdown of circTBX5 inhibits the expression of MyD88, a downstream of TLRs, and NF-κB signalling, which has been demonstrated in C28/12 cells [232].

LncRNA might represent a scaffold for molecules interacting with histones. For instance, a novel transcript of *ELDR* was found to mediate chondrocyte senescence. It binds to the *IHH* promoter sequence, mediating histone methylation and acetylation, which ultimately dysregulates hedgehog pathway and promotes senescence [233]. Furthermore, miRNA maturation is mediated by the methylation process. Liu et al. showed that miR-3591-5p promotes chondrocyte damage. M6a methylation of pri-miR-3591-5p is associated with promoted maturation. Demethylation leads to a maturation block, which suppresses OA [234]. Interestingly, *miRNA* gene sequences can also be methylated, which impacts their expression. According to a study by Papathanasiou et al., a regulatory sequence of *miR-140* is hypermethylated in OA chondrocytes. Additionally, a negative correlation has been found between miR-140-5p expression and *miR-140* methylation. Moreover, the authors observed increased methylation of the *miR-146a* promoter region in OA synoviocytes. The methylation level was also negatively correlated with miR-146a expression [235]. The genotypes of miR-146a SNP rs2910164 are differently associated with OA pathophysiology. The GG genotype is associated with the elevated expression of miR-146a, while the GC genotype is interestingly correlated with the increased expression of MMP-13, IL-6 and IL-1β [236]. Therefore, polymorphisms of ncRNA genes, as well as epigenetic modification (methylation), can impact their expression and function. Interestingly, the panel of miRNA might be used to distinguish OA patients. Baloun and colleagues demonstrated that miR-23a-3p, miR-146a-5p and miR-652-3p are elevated in the plasma of OA patients compared with healthy controls [237]. Consequently, ncRNA molecules might find use in the diagnostic process of OA in the future (Table 2).

## 5. Clinical Implications and Future Research

In this review, we focused on genetic variants associated with inflammation and catabolic factors, which have been associated with the pathogenesis of OA. To date, multiple polymorphisms have been identified to either increase or decrease the risk of OA in various populations. Selected alleles, genotypes or haplotypes could become genetic markers to distinguish a cohort with elevated risk of developing OA. Furthermore, described polymorphisms may correlate with transcriptional activity and, therefore, a certain genotype can be associated with elevated mRNA or protein levels of a particular cytokine or proteinase. These alleles or genotypes may require a different type of treatment or dosage. Multiple types of treatment agents are evaluated as potential disease-modifying drugs, such as inhibitors of MMPs, ADAMTS, IL-1, or IL-6, among many others [238]. For instance, in rheumatoid arthritis, the presence of the AA genotype of rs12083537 (*IL-6R* gene) was associated with a better response to tocilizumab, which targets IL-6 receptors [239]. However, a clinical trial by Richette et al. showed that there were no significant differences between tocilizumab and placebo in patients with hand OA [240]. Furthermore, genetic polymorphisms of TLRs, IL-1β, LY96, and TIRAP have been associated with a response to anti-TNFα or ustekinumab in psoriasis [241]. Nevertheless, similar studies in OA are yet to be performed. Recently, promising results have been published regarding TissueGene-C (TG-C), a treatment composed of human allogeneic chondrocytes together with the cells expressing TGF-β [242,243,244]. In addition, we have broadly discussed some of the epigenetic mechanisms involved in the pathogenesis of OA. ncRNAs could be implemented in the diagnosis and therapy of OA. As previously mentioned, the selected ncRNAs circulate in the plasma and their concentrations are higher in patients than in healthy controls. In addition, the use of miR-140 has also been investigated in the treatment. In animal models, Si et al. demonstrated that the intraarticular injection of miR-140 agomir was associated with slower disease progression, thicker cartilage, higher collagen II, and a lower expression of proteinases [245]. Moreover, current evidence suggests that some proteins associated with epigenetic regulation may become therapeutic targets, such as HDAC or EZH2. Nevertheless, the precise mechanisms of these enzymes in the pathogenesis needs to be investigated.

## 6. Conclusions

To conclude, OA is a chronic and progressive disease, which is associated with the activity of several pro-inflammatory pathways. In this review, we tried to demonstrate the importance of gene polymorphisms and epigenetics in the pathogenesis of OA. In the case of polymorphisms, we focused on variants identified in genes encoding pro-inflammatory mediators and degrading enzymes. To date, multiple SNPs have been associated with OA susceptibility. Selected alleles and genotypes could become clinical markers to distinguish a cohort with an elevated risk of developing OA. Nevertheless, conflicting results have been published in the case of certain genetic variants. This could be attributed to different sample sizes or populations. Moreover, some associations described in this article can be ethnically or geographically dependent, as a particular polymorphism may be much less common in selected populations. As a result, large studies across different populations would be required to evaluate the precise impact of a genetic variant. Furthermore, recent studies uncovered the extraordinary role of epigenetics in the regulation of gene expression and its contribution to disease pathogenesis. The regulation of proteins which take part in maintaining cartilage homeostasis or promote its degradation also contribute to the development of OA. Nevertheless, further research is required to fully understand the genetic and epigenetic mechanisms related to the progression of the disease. An interesting interplay between genetics and epigenetics should also be explored. In addition, new disease-modifying drugs are greatly needed in OA. More studies should investigate the targeting molecules involved in epigenetic regulation.

## Figures and Tables

**Table 1:** A list of selected polymorphisms associated with osteoarthritis in human-based studies.

Reference	Authors	Gene	Polymorphism	Findings	Joint	Population
[35]	Balbaloglu et al.	*TLR9*	rs187084	CC genotype was associated with an elevated risk of OA	Knee	Turkish
[36]	Zheng et al.	*TLR9*	rs187084	C allele and CC genotype were associated with an elevated risk of OA	Knee	Chinese
[37]	Yi et al.	*TLR9*	rs187084	Allele A is a risk factor	Hip	Chinese
[38]	Su et al.	*TLR9*	rs187084	−1486 TT genotype was associated with an elevated risk	Knee	Chinese
[39]	Stefik et al.	*TLR7*	rs3853839	GG genotype was more common in patients (28.4% vs. 13.5%)	Hip and Knee	Caucasians
[42]	Yang et al.	*TLR3*	rs3775296	C allele was associated with an elevated risk of OA	Knee	Chinese
			rs3775290	Genotype CT was associated with an elevated risk		
[39]	Stefik et al.	*TLR4*	rs4986790	Genotype AG was more common in patients	Hip and Knee	Caucasians
			rs4986791	Genotypes GG and CG were more common in patients		
[43]	Vrgoc et al.	*TLR10*	rs11096957	A/A genotype showed a predisposition to the disease	Hip	Croatian
[44]	Tang et al.	*TLR10*	rs11096957	T allele was associated with the risk of OA and was an indicator of the severity of the disease	Hip	Chinese
[47]	Han et al.	*RAGE*	82G/S	Allele S and genotype SS were associated with an increased risk of OA	Knee	Chinese
[73]	Stern et al.	*IL-1B*	5810G>A	Genotype AA was associated with hand OA and erosive hand OA	Hand	US Caucasoid population
[76]	Budhiparama et al.	*IL-1RN*	rs419598	IL-1RN*1 was associated with decreased risk IL-1RN*2 was associated with elevated risk	Knee	Caucasians
[81]	Hulin-Curtis et al.	*IL-18*	rs1946518	Wild-type allele was more common in patients	Knee	Caucasians
[105]	Yigit et al.	*IL-6*	rs1800795	Allele G and genotype GG were more common in patients	-	Turkish
[106]	Sun et al.	*IL-6*	rs1800795	Allele C was associated with the risk of OA	Knee	Chineese
[109]	Fernandes et al.	*IL-6*	rs1800796	Carriers of C allele have reduced susceptibility to OA	Knee/Hip	-
[111]	Yang et al.	*IL-6*	rs12700386	Allele C and CC genotype were associated with an elevated risk of OA	Knee	Chinese
[112]	Lu et al.	*TGF-β*	rs1982073	Carriers of at least one C allele were associated with increased risk of the disease	Knee	Chinese
[113]	Liu et al.	*TGF-β*	rs1800470	TT genotype was associated with the disease	-	Chinese
			rs1800469	T allele and TT genotype were associated with OA		
[135]	Liu et al.	* MMP-1 *	rs1799750	2G allele was associated with the disease	temporomandibular joint	Caucasians, Asians
[106]	Sun et al.	* MMP-13 *	rs2252070	Allele A was associated with the disease	Knee	Chinese
[153]	Ma et al.	* ADAMTS14 *	rs4747096	Allele G and GG genotype were associated with an elevated risk of OA	Knee	Chinese
[157]	Alimoradi et al.	* BCL-2 *	rs2279115	A recessive model CC vs. CA+AA was significantly associated with OA C allele was associated with OA	Knee	-
		* CXCL16 *	rs2277680	A dominant model GG+GA vs. AA was associated with OA G allele was associated with OA		
[158]	Wang et al.	*ITLN1/omentin*	rs2274908	Allele A was associated with OA susceptibility	Knee	Chinese
		* XCL2 *	rs4301615	Allele C was associated with OA susceptibility		
		* DOT1L *	rs3815308	Allele G was associated with OA susceptibility		
[162]	Hui et al.	* MGP *	rs1800802	GG genotype was associated with higher susceptibility to OAA recessive model GG vs. AG+AA and allele G were associated with an elevated risk of OA	Knee	Chinese

**Table 2:** A summary of selected epigenetic mechanisms and the role of selected enzymes and molecules associated with epigenetics in OA.

Epigenetic Mechanism	Selected Findings in OA		References
DNA Methylation	Hypomethylated CtBP, IL-16, and IL-6 promoters. Hypermethylated COL9A1 promoter.		[174,175,177,181]
Histone modifications (Acetylation, Methylation, HDAC Inhibitors)	Elevated HDAC1 and HDAC2 in OA-derived chondrocytes, which are involved in the suppression of collagen and aggrecan expression. Beneficial effects of HDAC inhibitors. Chondrocyte stimulation with IL-1 associated with H3K4 methylation of iNOS and COX2 promoters. Inhibition of H3K9 promotes MMP expression in mouse chondroprogenitor cells. Association between EZH2 (histone methyltransferase) and pro-inflammatory and catabolic factors.		[189,199,200,201,208,210,212,213]
Non-Coding RNA	Expression in OA	Role	
miR-17	Decreased in DMM-induced OA mice	Suppression of NOS2, ADAMTS5, MMP3, MMP13 in cultured-mice chondrocytes	[225]
miR-17-5p	Decreased in OA cartilage tissues	Targets EZH2 and inhibits 1β-induced ECM degradation	[229]
miR-149	Decreased in OA patients	Targets VCAM-1 and suppresses inflammation in animal model	[226]
miR-34a-5p	Elevated in OA patients	Mimic decreased the expression of COL2A1 and ACAN, and promoted MMP13, ADAMTS5, IL-1β in chondrocytes	[227]
miR-222	Decreased in OA chondrocytes	Targets HDAC4 ad inhibits MMP-13 in DMM-induced mice	[228]
circSCAPER	Elevated in OA cartilage tissues	Targets miR-140-3p and regulates EZH2 expression	[230]
circTBX	Elevated in OA cartilage tissues	Targets miR-558 and positively regulates MyD88	[232]

## Data Availability

Not applicable.
